# Preservation of Meat for Ten Years

**Published:** 1892-02

**Authors:** 


PRESERVATION OF MEAT FOR TEN YEARS.—A gentleman in New York has just tested the result of preserving a turkey in a refrigerator for ten years. This time having elapsed, the fowl was removed from the refrigerator a short time ago, and after being properly cooked, was eaten by a party of well-known gentlemen. While putrefactive changes seem to have been entirely avoided, it was found that the meat was practically tasteless, and had lost all of its characteristic flavor.—*Scientific American*, December 19, 1891. (*Climatologist.*)

